# Supplementary material for: Gamma-aminobutyric acid elicits H2O2 signalling and promotes wheat seed germination under combined salt and heat stress
Source: PeerJ. 2024 Sep 18;12:e17907. doi: 10.7717/peerj.17907 (PMC11416083; doi:10.7717/peerj.17907)
Supplement: Supplemental Information 6 [file peerj-12-17907-s006.docx]

# RNA integrity verification electrophoretic profiles


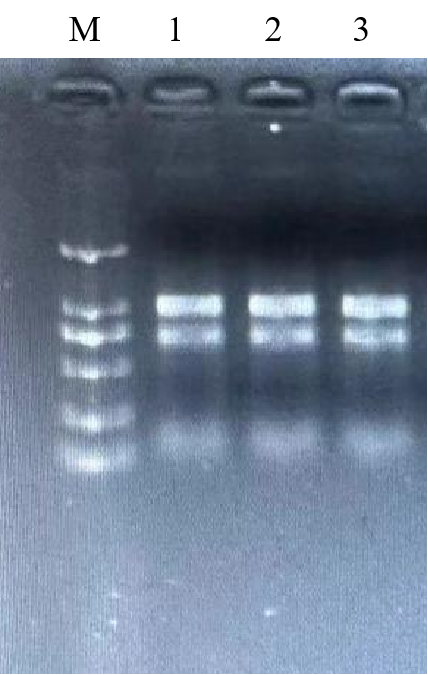


Figure 1 RNA agarose gel electrophoresis

M: DL 2000 Maker; 1, 2, 3: RNA samples from three biological replicates of wheat seeds incubated for 8 h at 0 mM NaCl+20°C, respectively

# Results of UV spectral analysis of RNA purity and concentration


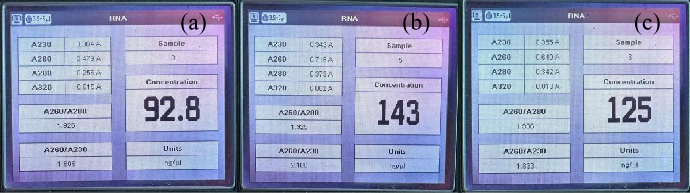


Figure 2 Contamination assessment and purity of RNA (A260/A280).

a,.b,,c: RNA samples from three biological replicates of wheat seeds incubated for 8 h at 0 mM NaCl + 20°C, respectively

# Wheat primer specificity screen

a
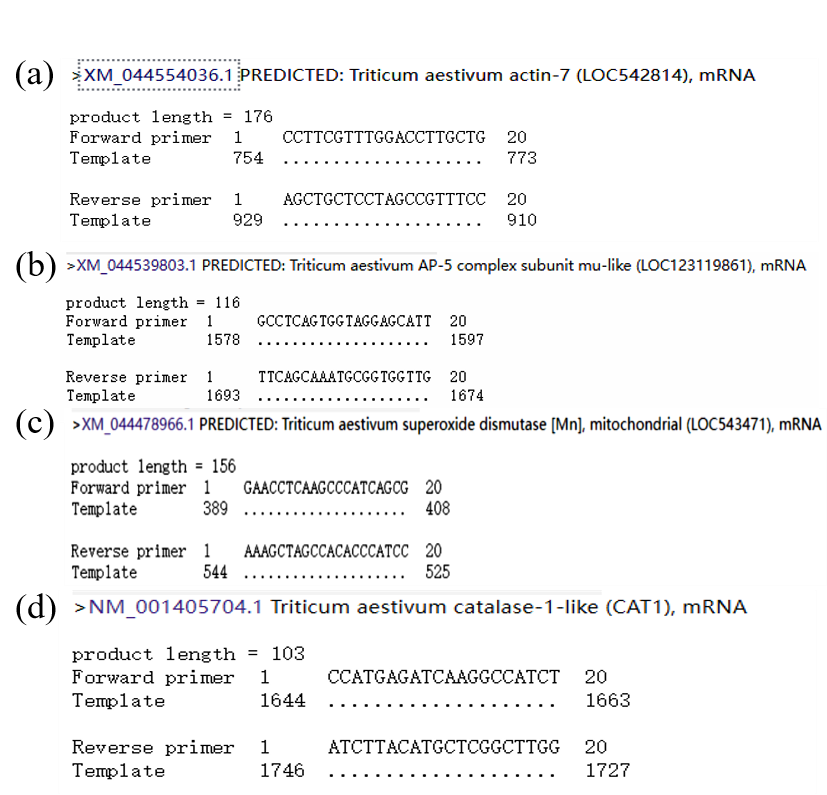


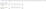


Figure 3 Specificity screen of primers.

a：*actin*；b：*CJ705892*；c：*MnSOD*；d：*CAT*

# RNA inhibition test results

Figure 4 Cq values of qRT-PCR tests for detecting RNA inhibition

Note: Control is the Cq value of qRT-PCR using cow somatic cell cDNA as template and β-actin internal reference gene; control + wheat seed RNA is the RNA of wheat seeds added to the control qRT-PCR system (wheat seeds were incubated for 8 h at 0 mM NaCl + 20°C)


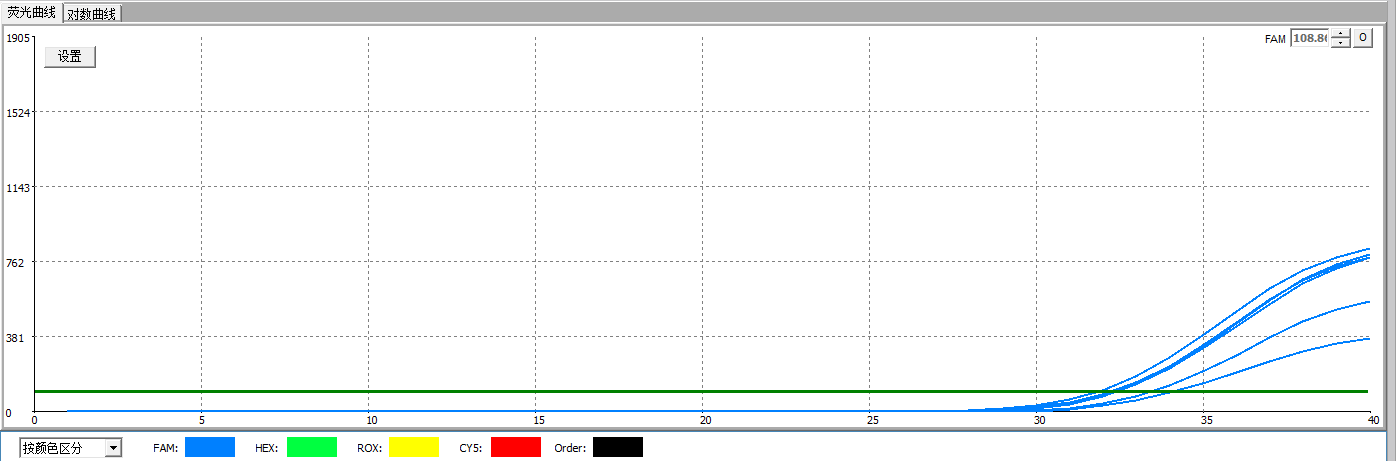

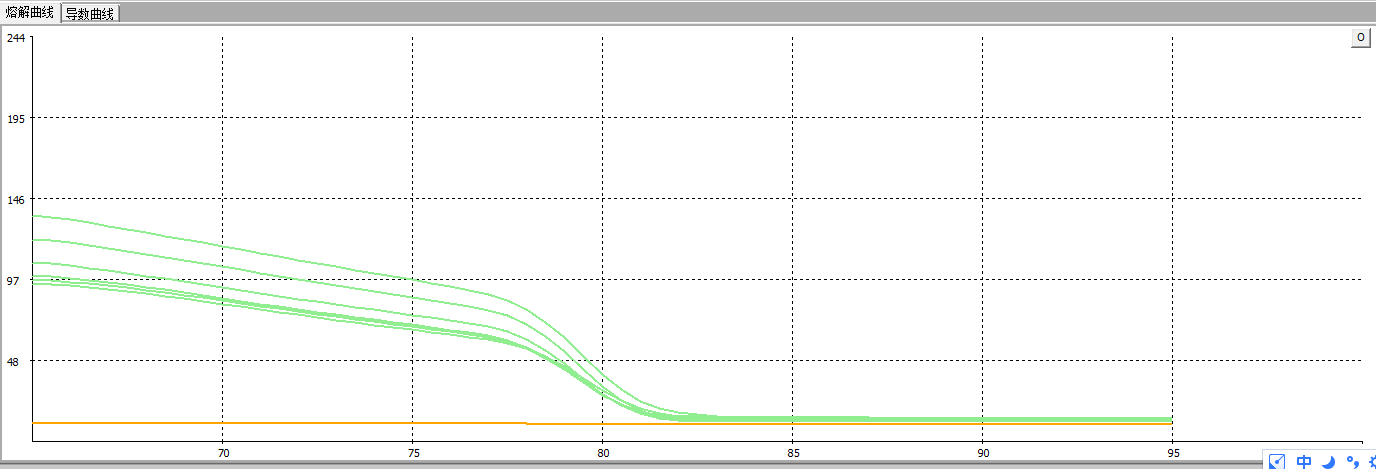

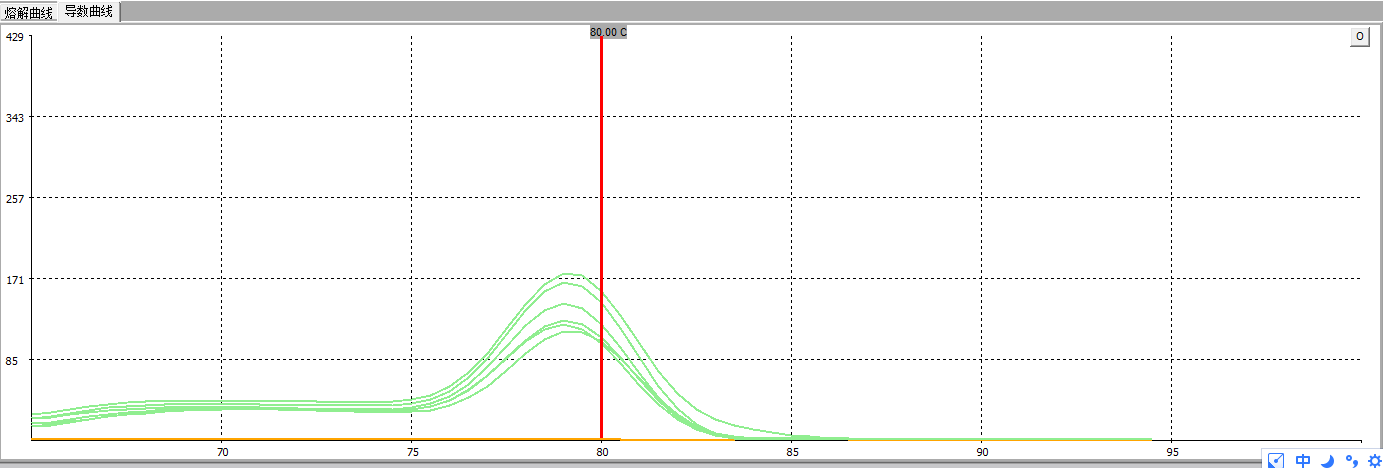


(a)

(b)

(c)

Figure 5 qRT-PCR detection of RNA inhibition in wheat seeds

a: fluorescence curve; b: melting curve; c: derivative curve

# Standard curves for different primers

a
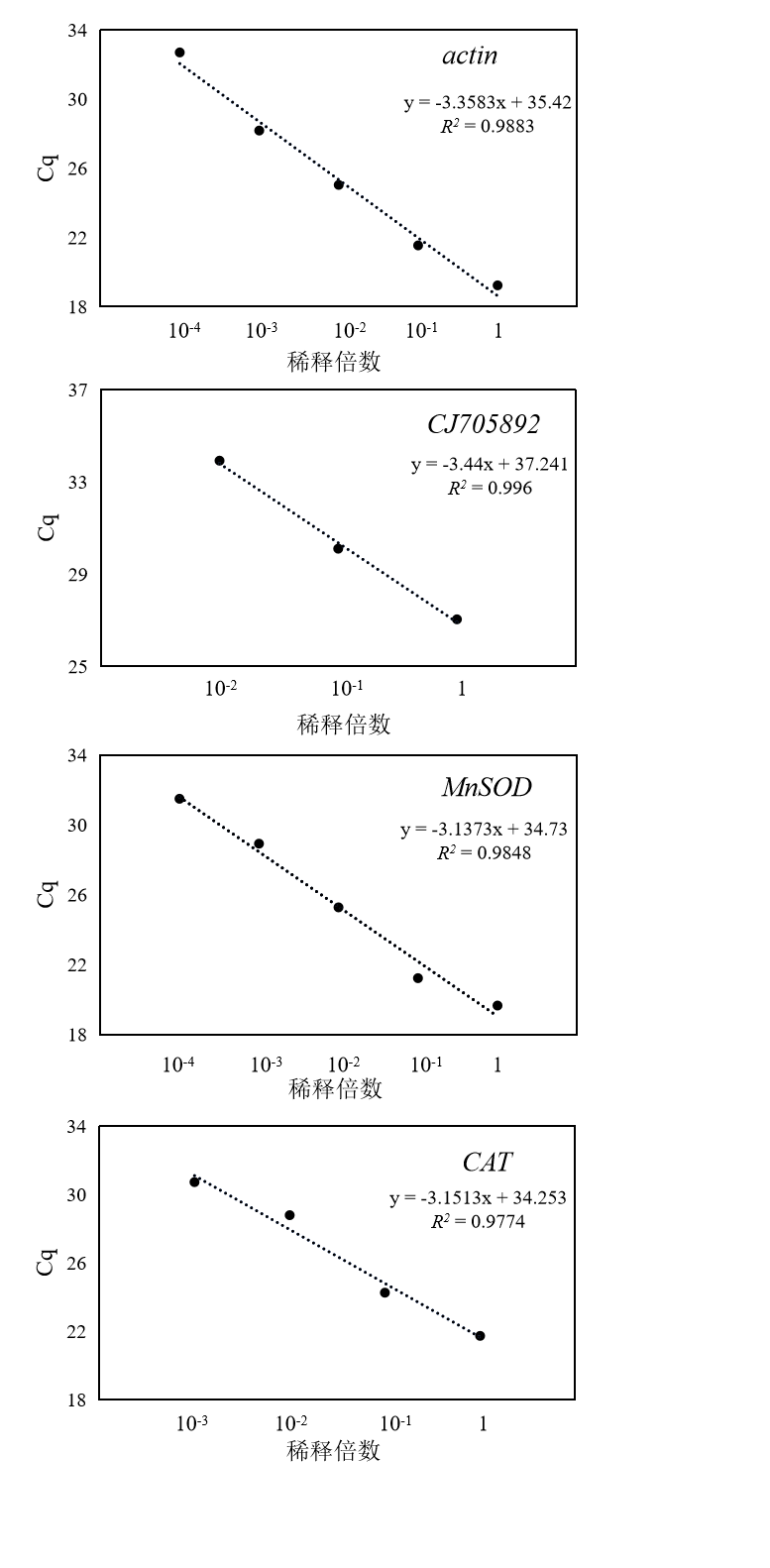


Figure 6 Standard curves for different primers

# Evidence of detection limits for different primers


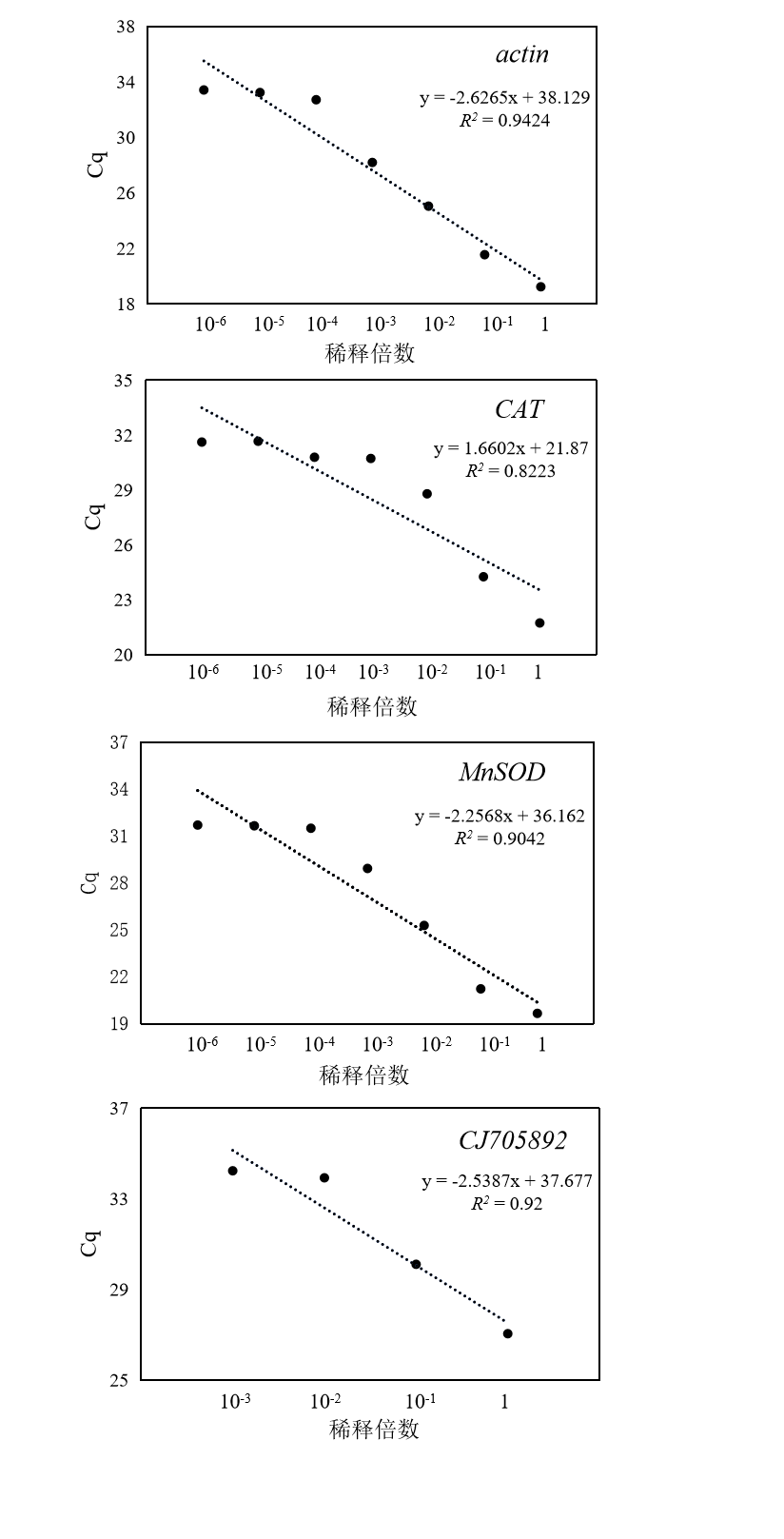


Figure 7 Standard curves for detection limits of different primers


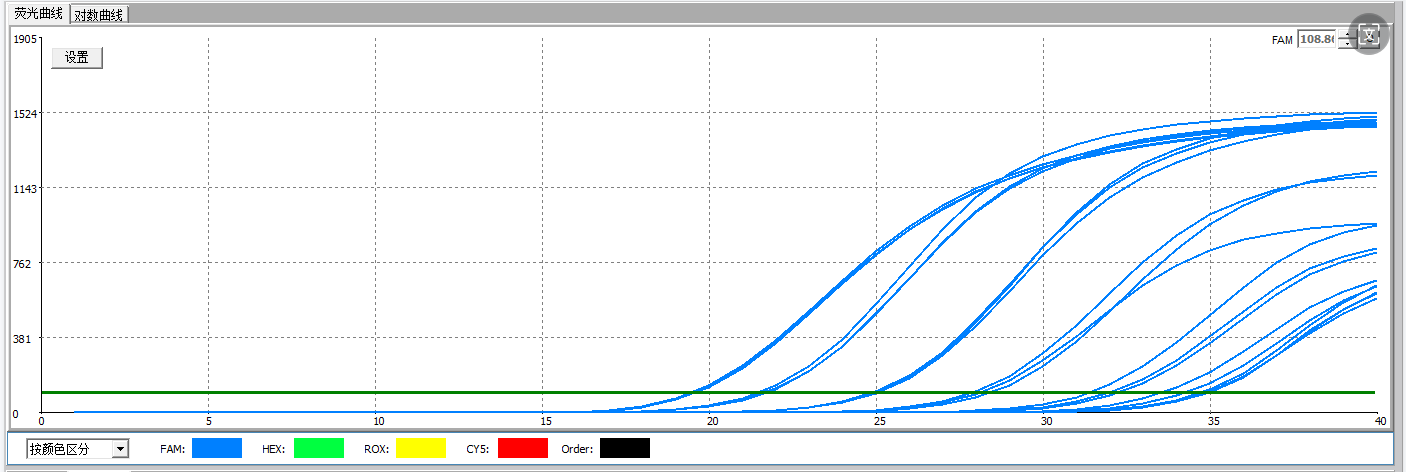

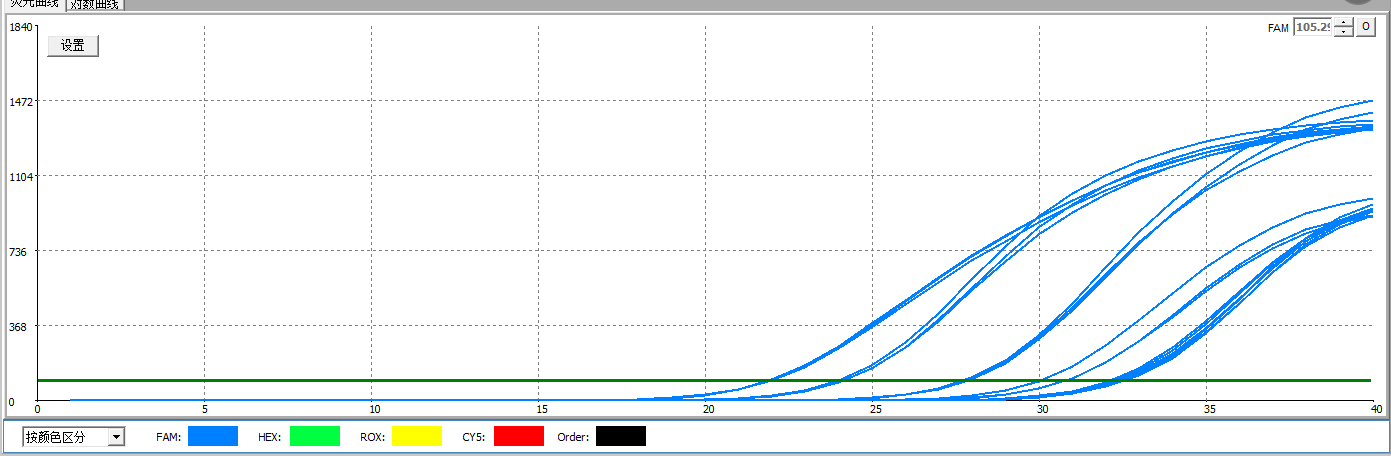


CAT


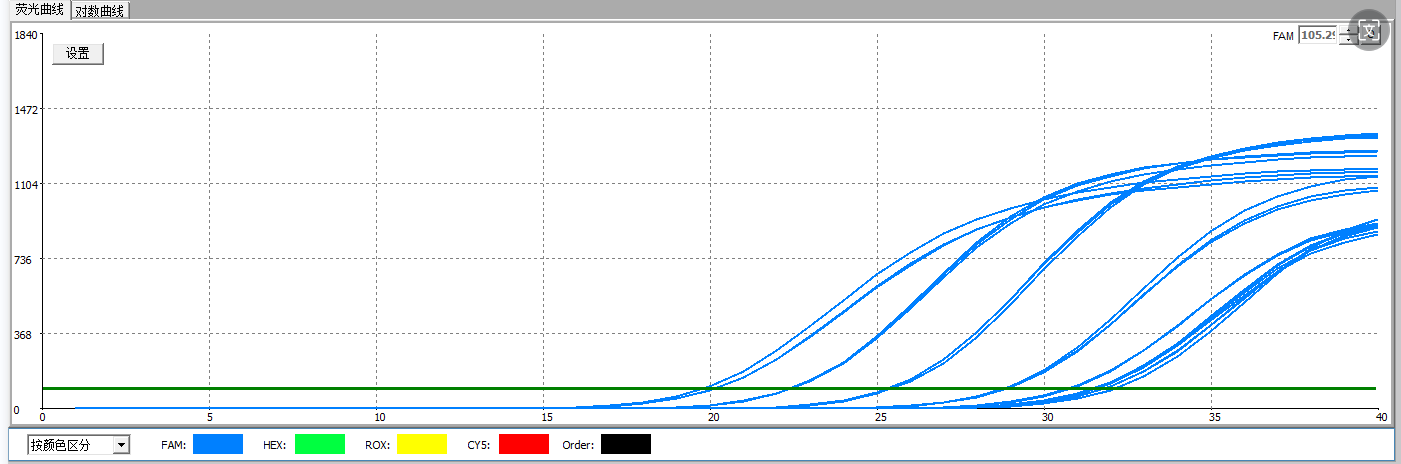

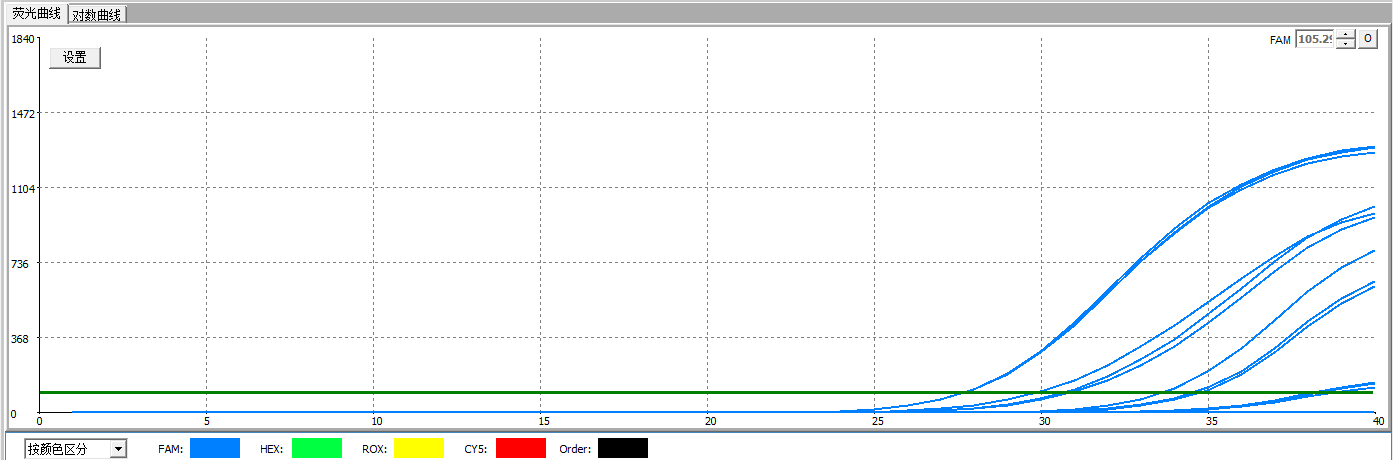


(b)

(d)

(a)

(c)

Figure 8 Fluorescence curves for different primer detection limits

a：*actin*；b：*CAT*；c：*MnSOD*；d：*CJ705892*


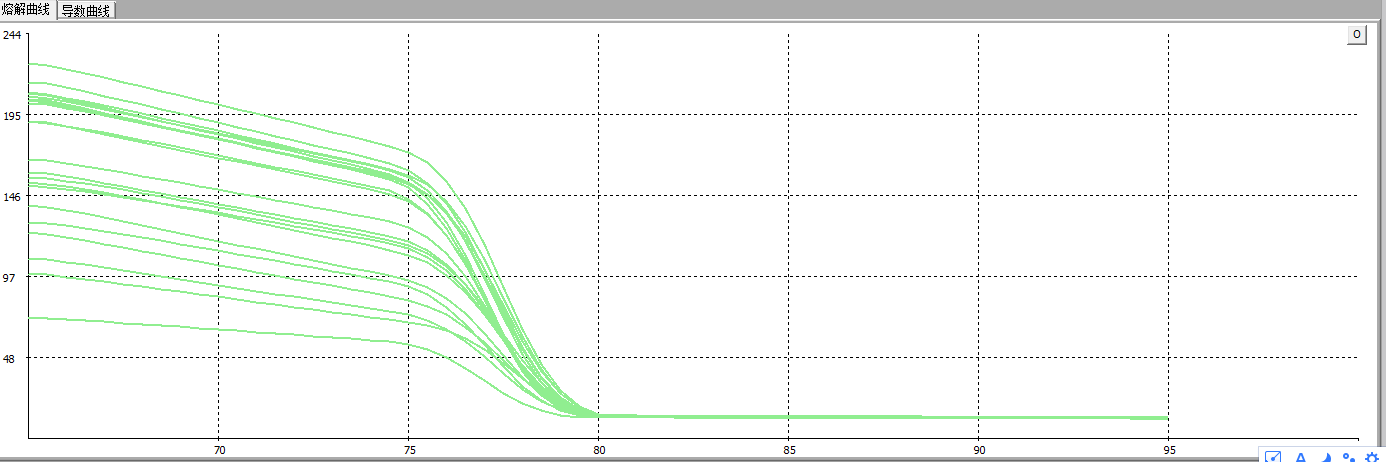

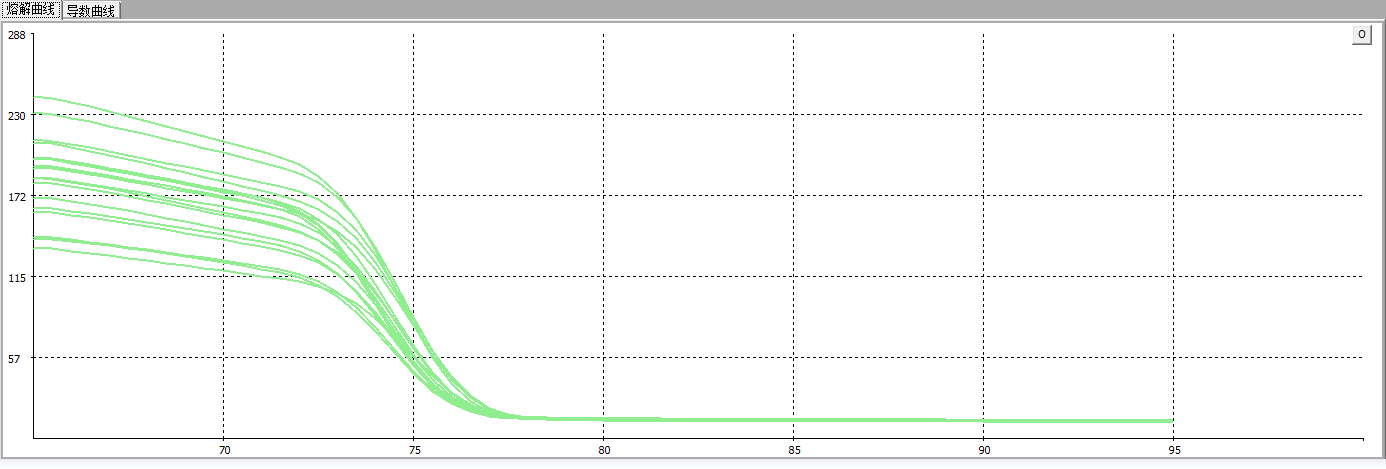

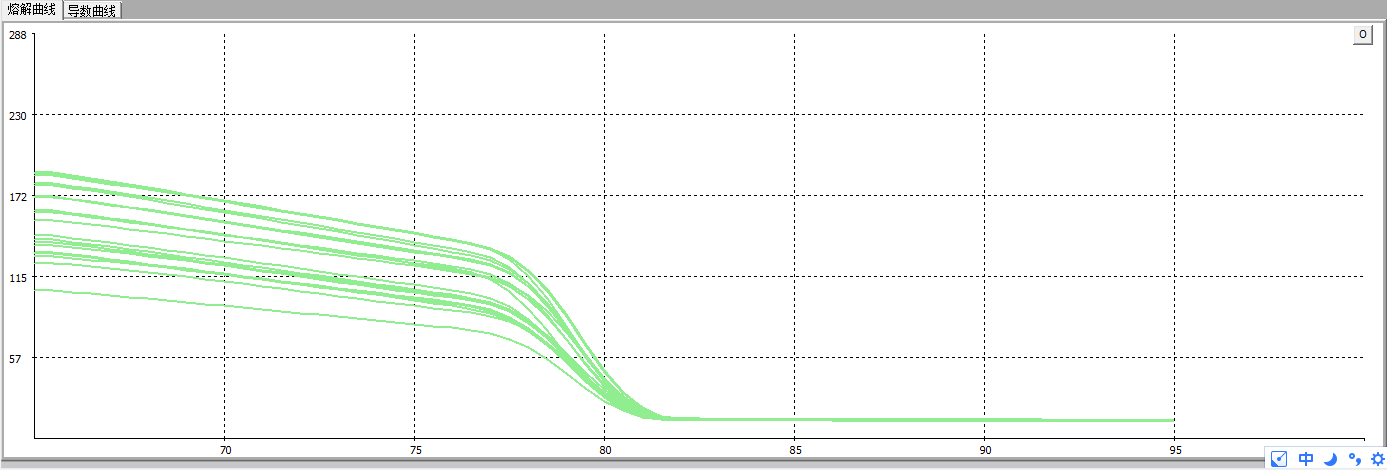

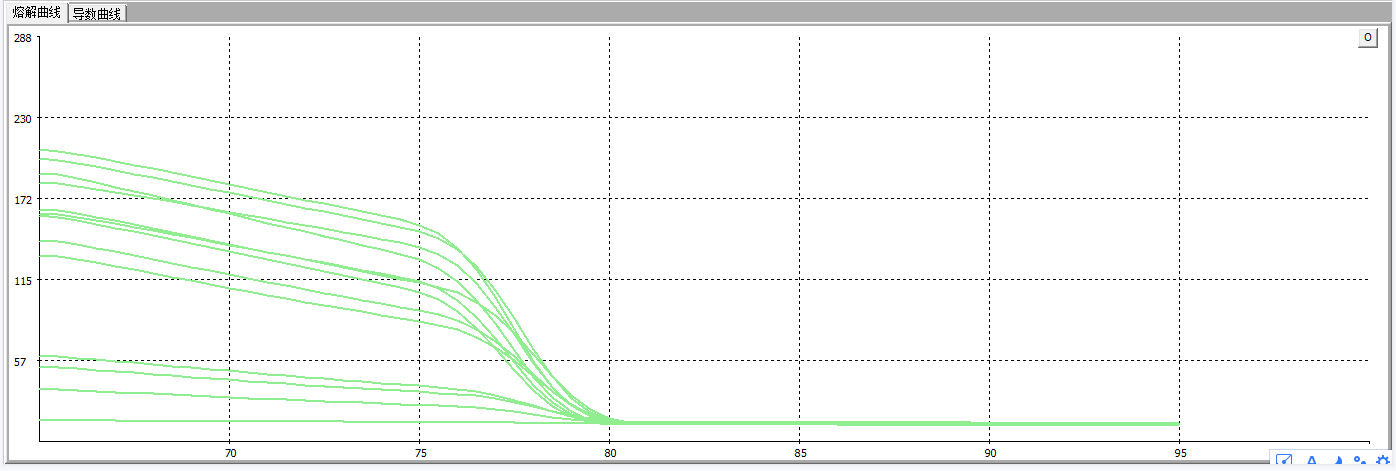


(a)

(d)

(c)

(b)

Figure 9 Melting curves with different primer detection limits

a：*actin*；b：*CAT*；c：*MnSOD*；d：*CJ705892*


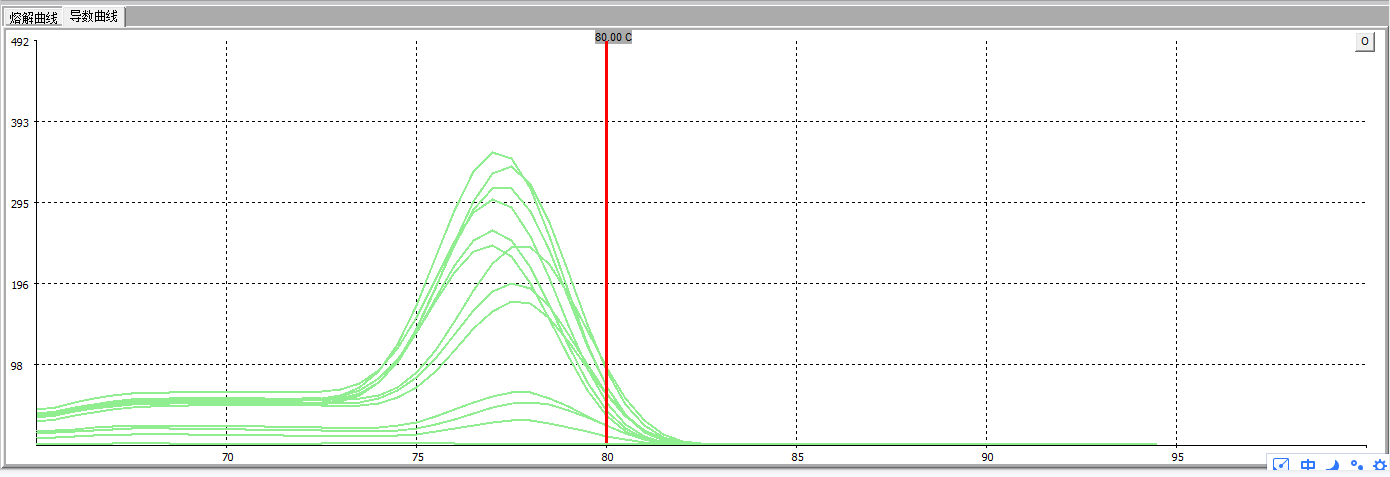

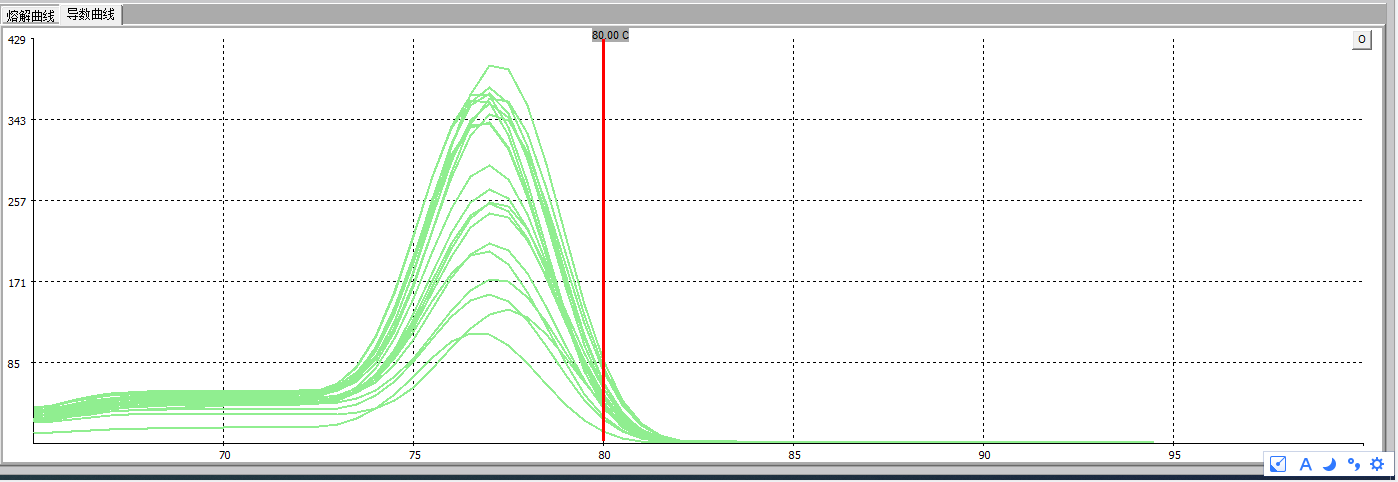

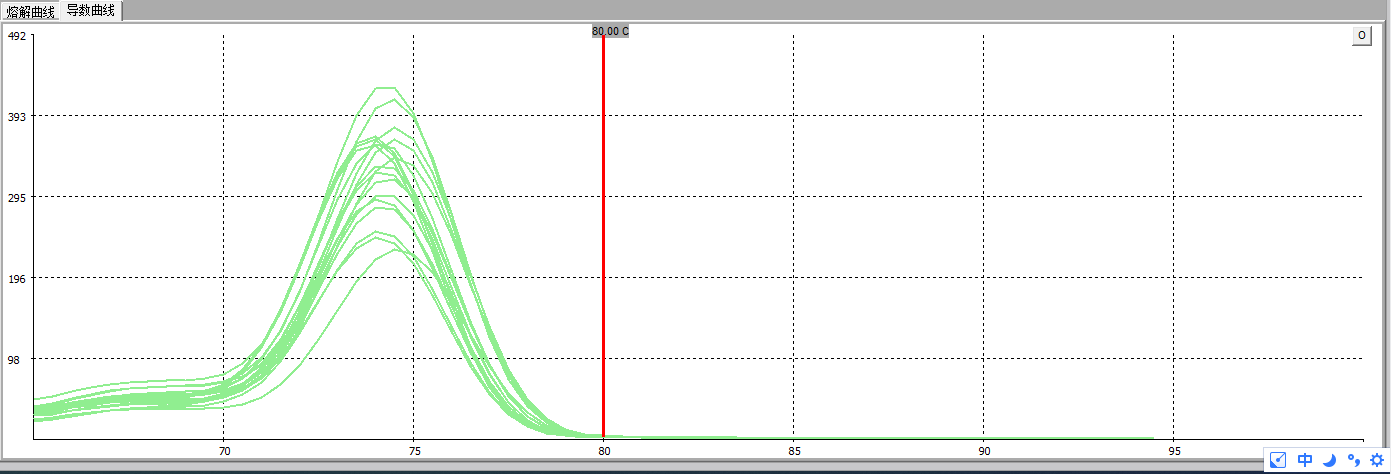

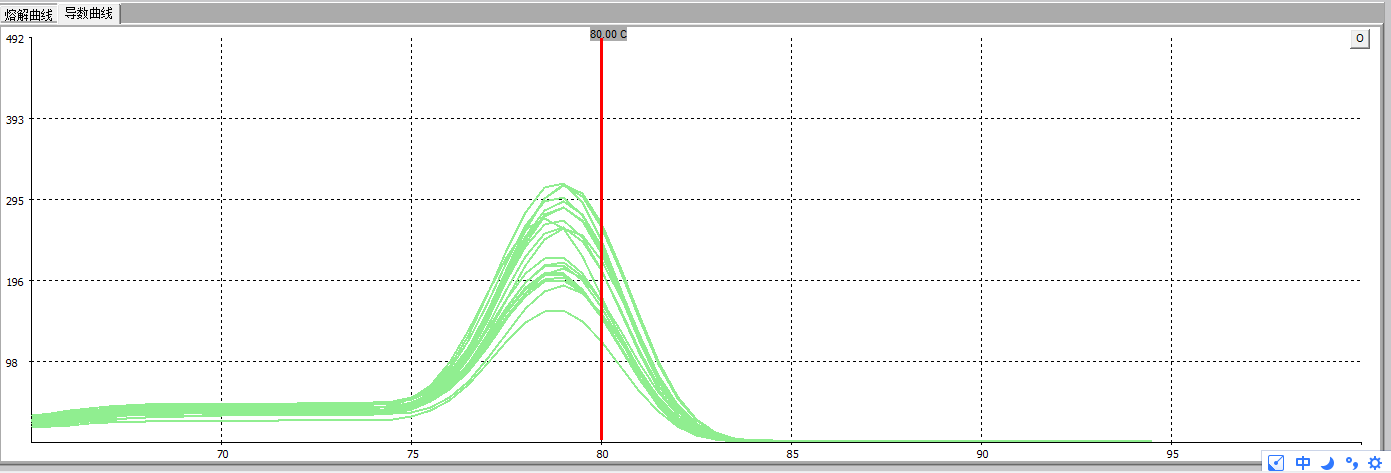


(a)

(d)

(c)

(b)

Figure 10 Derivative curves for detection limits of different primers

a：*actin*；b：*CAT*；c：*MnSOD*；d：*CJ705892*

# Screening of reference genes


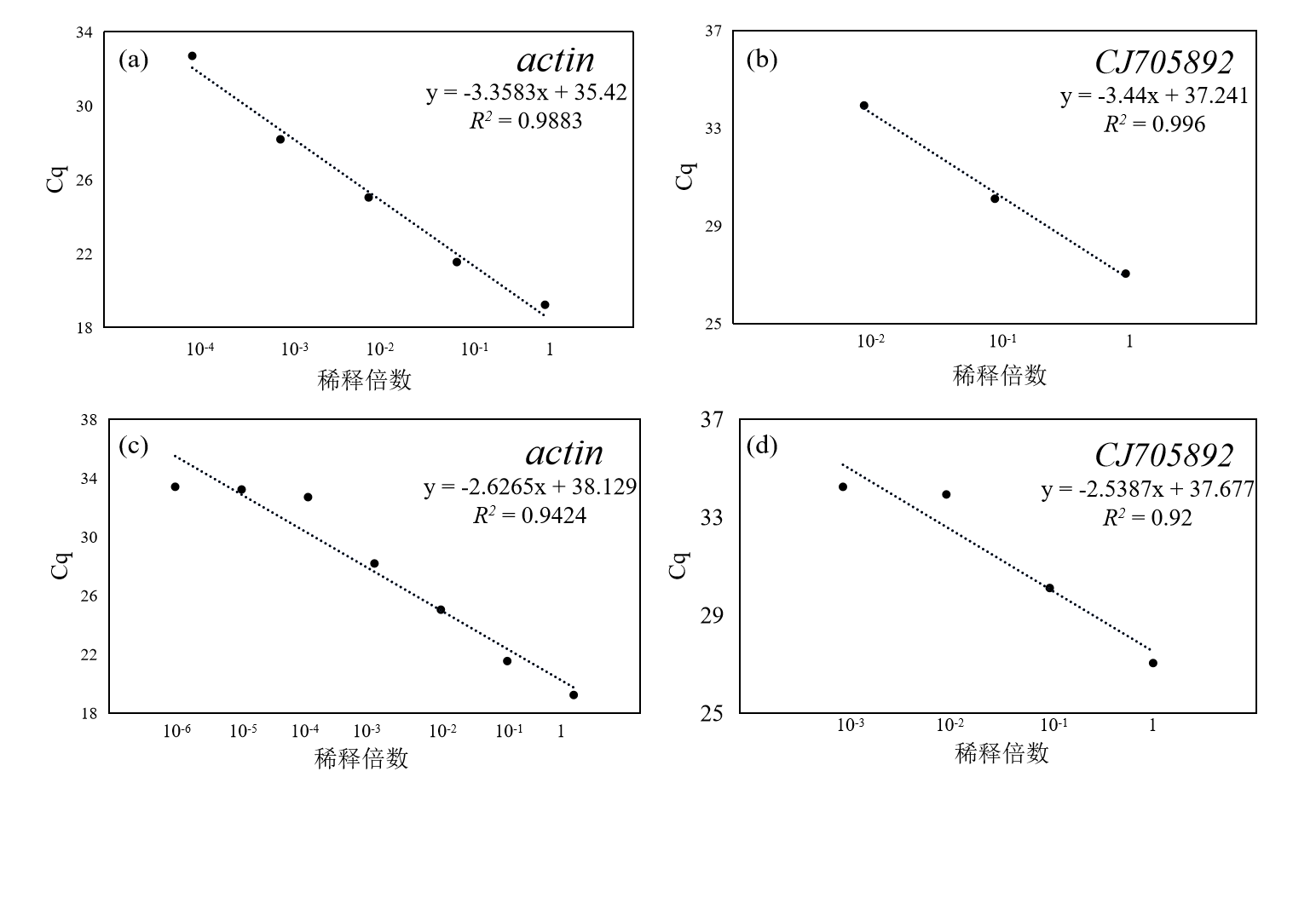


啊啊啊啊啊Figure 11 Reference gene screening evidence

a, b: Standard curves for *actin* and *CJ705892* used to calculate amplification efficiency;

c, d: Evidence of detection limits for *actin* and *CJ705892*

The amplification efficiency slopes of the two reference genes, *actin* and *CJ705892*, are -3.3583 and -3.44, respectively, as can be seen in plots a and b. Both are slightly higher than the ideal slope of -3.3, which is within the acceptable standard range of amplification efficiencies (90-110%) according to the amplification efficiency calculation formula. In addition, the *actin* gene in plots c and d exhibits lower cycling threshold (Cq) values at a range of dilution concentrations, enabling efficient detection at lower template concentrations (10^-4^) and thus exhibiting a wider dynamic detection range. In contrast, *CJ705892* reached the limit of detection at a dilution concentration of 10^-2^ and could no longer be effectively detected, and was unable to detect Cq values at lower dilutions (10^-4^), indicating a limited dynamic range of detection. Therefore, we chose *actin* as the internal reference gene for the calculation.

# Different primer sequences

Table 1 Primer Sequence List

| S/NO | Gene_  name | Gene ID | Amplifier length (BP) | Forward | Reverse |
| --- | --- | --- | --- | --- | --- |
| 1 | *Actin* | XM_044554036.1 | 176 | CCTTCGTTTGGACCTTGCTG | AGCTGCTCCTAGCCGTTTCC |
| 2 | *MnSOD* | XM_044478966.1 | 156 | GAACCTCAAGCCCATCAGCG | AAAGCTAGCCACACCCATCC |
| 3 | *CAT* | NM_001405704.1 | 103 | CCATGAGATCAAGGCCATCT | ATCTTACATGCTCGGCTTGG |
| 4 | *CJ705892* | XM_044539803.1 | 116 | GCCTCAGTGGTAGGAGCATT | TTCAGCAAATGCGGTGGTTG |
